# Genetic Analysis and Fine Mapping of *ZmGHT1* Conferring Glufosinate Herbicide Tolerance in Maize (*Zea mays* L.)

**DOI:** 10.3390/ijms231911481

**Published:** 2022-09-29

**Authors:** Jianxi Bao, Yuexin Gao, Yanan Li, Suowei Wu, Jinping Li, Zhenying Dong, Xiangyuan Wan

**Affiliations:** 1 Zhongzhi International Institute of Agricultural Biosciences, Shunde Graduate School, Research Center of Biology and Agriculture, School of Chemistry and Biological Engineering, University of Science and Technology Beijing, Beijing 100024, China; 2 Beijing Engineering Laboratory of Main Crop Bio-Tech Breeding, Beijing International Science and Technology Cooperation Base of Bio-Tech Breeding, Beijing Solidwill Sci-Tech Co., Ltd., Beijing 100192, China

**Keywords:** maize, herbicide tolerance, fine mapping, structural variation, candidate gene

## Abstract

Weed interference in the crop field is one of the major biotic stresses causing dramatic crop yield losses, and the development of herbicide-resistant crops is critical for weed control in the application of herbicide technologies. To identify herbicide-resistant germplasms, we screened 854 maize inbreed lines and 25,620 seedlings by spraying them with 1 g/L glufosinate. One plant (L336R), possibly derived from a natural variation of line L336, was identified to have the potential for glufosinate tolerance. Genetic analysis validated that the glufosinate tolerance of L336R is conferred by a single locus, which was tentatively designated as *ZmGHT1*. By constructing a bi-parental population derived from L336R, and a glufosinate sensitive line L312, *ZmGHT1* was mapped between molecular markers M9 and M10. Interestingly, genomic comparation between the two sequenced reference genomes showed that large scale structural variations (SVs) occurred within the mapped region, resulting in 2.16 Mb in the inbreed line B73, and 11.5 kb in CML277, respectively. During the fine mapping process, we did not detect any additional recombinant, even by using more than 9500 F_2_ and F_3_ plants, suspecting that SVs should also have occurred between L336R and L312 in this region, which inhibited recombination. By evaluating the expression of the genes within the mapped interval and using functional annotation, we predict that the gene *Zm00001eb361930*, encoding an aminotransferase, is the most likely causative gene. After glufosinate treatment, lower levels of ammonia content and a higher activity of glutamine synthetase (GS) in L336R were detected compared with those of L336 and L312, suggesting that the target gene may participate in ammonia elimination involving GS activity. Collectively, our study can provide a material resource for maize herbicide resistant breeding, with the potential to reveal a new mechanism for herbicide resistance.

## 1. Introduction

Weeds are the major constraint to crop production when compared with other foes, such as pathogens and animal pests, in most cropping systems, producing about 43% of global losses annually [1]. There are more than 35,000 weed species distributed widely around the world, of which about 8000 species are considered troublesome for agriculture [2]. In China, there are more than 1400 weed species having harmful effects for crops [3], and more than 3 million tons of grain losses are caused by weeds each year [4]. While in the United States, maize yield loss due to weed interference is about 50%, causing an estimated annual economic loss of USD 26.7 billion [5].

Because of the high efficacy, low labor requirements, and low cost, herbicides were quickly accepted and adopted by farmers once they were introduced in the late 1940s and early 1950s [6]. Since the introduction of herbicides into China in the late 1950s, they have currently become the second-most used class of pesticides, following insecticides [7]. However, the intense use of herbicides in the crop field has imposed widespread selection pressure, and herbicide-resistant weeds have quickly evolved, decreasing the number of effective and available herbicides for problematic weeds are over time. Currently, 267 (154 dicots and 113 monocots) herbicide resistant weed species have been reported in 72 countries, and weeds have evolved resistance to 21 of the 31 known herbicide sites of action and to 165 different herbicides [8]. For the efficient control of weeds, creating new herbicides that crops, but not weeds, can tolerate, or developing crops that can tolerate herbicides are crucial for weed control and crop production. Due to the long experimental period and high development costs, new herbicides cannot be rapidly commercialized, and there is a risk that the evolutionary rate of herbicide-resistant weeds will possibly exceed the rate of new herbicide discovery [9,10]. Developing herbicide resistant crops that can reduce phytotoxicity after herbicide application is a valuable and practicable alternative for weed management in agriculture.

Depending on the knowledge regarding herbicide-resistant plants, proteins, especially some enzymes, involved in the regulation of key metabolic processes in plant development are the main molecular targets [11,12]. For example, 5-enolpyruvylshikimate-3-phosphate synthase (EPSPS; EC 2.5.1.19) belongs to a kind of amino acid synthase which is a key enzyme in the shikimate pathway for the biosynthesis of aromatic amino acids and phenolics [13,14]. EPSPS is the main target of glyphosate, which is a broad-spectrum, post-emergent herbicide that is most widely used for weed control. Glyphosate can bind to EPSPS irreversibly and compete with the normal substrate phosphoenolpyruvate (PEP) [13,14]. Shikimate will be excessively accumulated once the EPSPS activity is disrupted, and the synthesis of secondary metabolites, including some plant hormones via aromatic amino acids, are also obstructed; these in turn cause disorders in plant growth and ultimately, plant death [13,14]. The mutation of maize EPSPS with T102I and P106S resulted in insensitivity to glyphosate inhibition, but high affinity for PEP has been successfully used commercially for developing glyphosate-resistant maize [15,16]. In addition, investigations of some glyphosate resistant weeds showed that EPSPS gene duplication occurred, and both the transcript and protein accumulation of EPSPS have a linear correlation with the EPSPS genomic copy number, indicating that enough uninhibited EPSPS can also confer glyphosate resistance [11].

Due to the wide application of glyphosate, weed resistance to glyphosate was increasingly reported. A total of 56 glyphosate resistant weed species (from 344 unique cases) have been discovered; in contrast, only 5 glufosinate resistant weed species (from 9 unique cases) have been described [8]. Glufosinate is another non-selective, post-emergent herbicide which is less widely used than glyphosate, and GS (EC 6.3.1.2) is its main target. GS catalyzes the incorporation of ammonia with glutamate to form glutamine, and it is thus essential for plant nitrogen metabolism [17,18]. Glufosinate competes for the glutamate binding site and irreversibly inhibits the activity of GS; the sensitive plants will accumulate excess ammonia quickly after the application of glufosinate, triggering a series of metabolic abnormalities which ultimately induce plant death [17,18,19]. In higher plants, there are two major GS isoforms, namely cytosolic GS1 and plastidic GS2 [20]. A mutation of D171N in the Italian ryegrass *GS2* gene was first reported as correlating with glufosinate resistance [21]. Recently, A novel point mutation (S59G) was identified in the *GS1* gene of the *Eleusine indica* glufosinate-resistant population, and the transformed rice with the mutant *GS1* gene is resistant to glufosinate [22].

Maize has become the world’s second most cultivated crop (FAOSTAT, https://www.fao.org/faostat, accessed on 17 February 2022). Weed control via herbicides and the development of new herbicide resistant varieties are both urgently needed for maize production. Herbicide resistant varieties can be obtained by transgenic technologies, or by the mutagenesis of crop genomes and the screening of mutants with enhanced herbicide resistance. There have been 128 recorded transgenic events regarding herbicide-tolerant maize reported in the International Service for the Acquisition of Agri-biotech Applications (ISAAA, https://www.isaaa.org/, accessed on 31 May 2022). However, the public perception and strict regulatory policies developed over centuries gave limited the application of genetically modified (GM) crops [23]. Herbicide-resistance obtained through natural variation or artificial mutagenesis are valuable in breeding herbicide resistant crops, and these methods have been reported in rice and maize [24,25,26,27]. In this study, we screened the maize germplasms kept in our institute for glufosinate resistance, and we mapped the *ZmGHT1* conferring glufosinate herbicide tolerance in maize using a segregation population derived from the identified resistant line L336R and a sensitive line L312. The *ZmGHT1* was located in a region of the 2.16 Mb interval of maize chromosome 8 (Chr8), and the candidate gene was predicated, which will provide genetic basis for glufosinate tolerant maize breeding.

## 2. Results

### 2.1. Isolation and Response of L336R to Glufosinate

**Figure 1:**
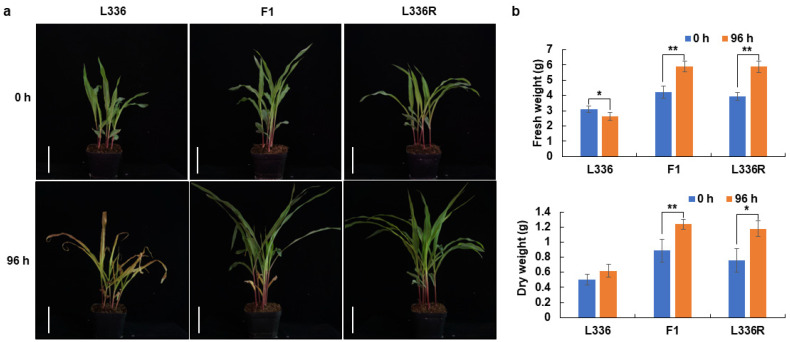
Responses to glufosinate among L336, L336R, and the F_1_ plants. (**a**) Visual injury after glufosinate treatment; scale bar = 10 cm; (**b**) above ground biomass measurement. * indicates *p* < 0.05 and ** indicates *p* < 0.01, respectively.

A total of 854 maize inbred lines with different genetic backgrounds were kept in our laboratory [28]. To isolate the glufosinate tolerant germplasms, 30 seedlings per line were treated with 1 g/L glufosinate, and finally one single plant (designated as L336R), derived from L336, was suspected to possess glufosinate tolerance. L336R was self-pollinated and the derived seedlings were used to evaluate the response to glufosinate. By treating it with 1 g/L glufosinate, progressive injury occurred in L336; the leaves started to turn yellow at 48 h after glufosinate treatment (AGT), and the whole plant dried out and died at 96 h (Figure 1a and Figure S1). However, no visual injury was observed in L336R during the glufosinate treatment (Figure 1a and Figure S1). The above ground biomass measurement showed that the fresh weight of L336 decreased, while that of L336R increased significantly at 96 h AGT (Figure 1b). For the dry weight, L336 showed a slight increase, but a nearly 55% increase occurred in L336R at 96 h AGT (Figure 1b). 

To further evaluate the glufosinate resistance, different doses of glufosinate were applied to L336R at the three-leaf stage, and the phenotypes were observed at 96 h AGT. The first leaves of the seedlings turned yellow, and seedling growth was obviously inhibited under 5 g/L glufosinate, but the whole plants shrank and began to die after treatment with 8 g/L glufosinate (Figure S2). Such observations indicate that L336R should be tolerant to glufosinate at doses of no more than 5 g/L.

### 2.2. Inheritance of ZmGHT1

L336R was first crossed with L336, and it was observed that the F_1_ plants behaved in a similar manner to L336R in being resistant to glufosinate after spraying with 1 g/L glufosinate (Figure 1). L336R was then crossed with a glufosinate sensitive line L312, and 192 F_2:3_ families from self-pollinated F_2_ plants were systematically screened 96 h after glufosinate treatment. A total of 47 and 46 families were identified as homozygously resistant and susceptible to glufosinate, respectively, and 99 families were heterozygous, as both glufosinate susceptible and resistant progenies were obtained (Table 1). The responses of the F_2:3_ families to glufosinate were then validated to fit the segregation ratio of 1:2:1, indicating that *ZmGHT1* was segregated from a single dominant locus conferring resistance to glufosinate. The remaining 9500 F_3_ seedlings generated from 30 F_2_ plants heterozygous for *ZmGHT1* were also examined for glufosinate resistance, and 7057 resistant and 2443 susceptible plants were detected, respectively. Chi-squared testing indicated that the segregation fitted the ratio of 3:1 (χ^2^ = 2.596 < χ^2^_0.05_ = 3.841), further suggesting that *ZmGHT1* should be behaved as a single dominant nuclear gene.

### 2.3. Genetic Mapping of ZmGHT1

**Figure 2:**
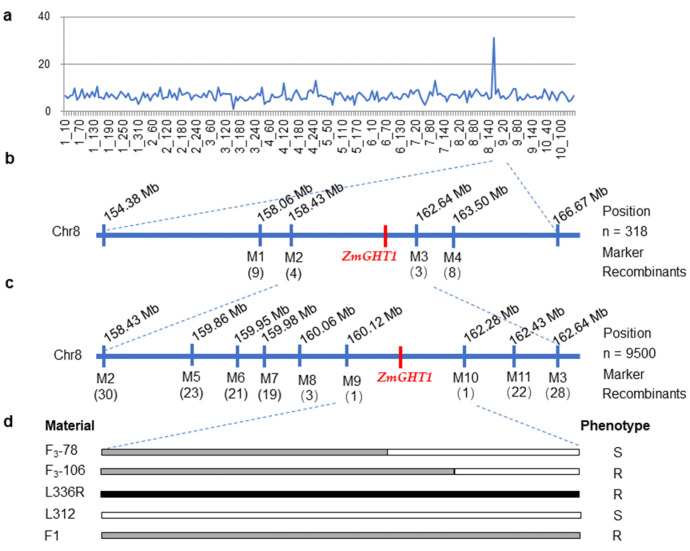
Fine mapping of *ZmGHT1*. (**a**) Polymorphic ratios between tolerant and sensitive DNA pools detected by the Maize 60K SNP chip; (**b**) primary mapping of *ZmGHT1*, and the *ZmGHT1* region was delimited to a 4.21 Mb interval between M2 and M3 in Chr8; (**c**) fine mapping of *ZmGHT1*, and the *ZmGHT1* was mapped between M9 and M10; (**d**) illustration of the key recombinants and phenotype determination. Black bars represent chromosomal fragments derived from L336R, white bars represent the fragments derived from L312, and gray bars indicate the heterozygous regions.

F_2_ plants derived from the cross of L336R and L312 were used for primary mapping. As a first step, two bulked DNA pools from ten tolerant and ten sensitive F_2_ individuals were genotyped using the Maize 60K SNP chip, respectively. Higher polymorphic ratios were detected between 154.38 and 166.67 Mb on Chr8 (Figure 2a); *ZmGHT1* was therefore assigned to Chr8, and SSR markers were developed within this region. In total, 11 polymorphic and co-dominant molecular markers were used for mapping *ZmGHT1* in this study (Table 2). 

Using molecular markers of M1 to M4 and 318 F_2_ plants with known phenotypes, *ZmGHT1* was primarily located in a 4.21 Mb region between markers M2 and M3 (Figure 2b). To further narrow the mapped region of *ZmGHT1*, 7 additional molecular markers (M5 to M11) were used, and although a large population possessing up to 9,500 F_3_ plants were examined, only 1 resistant plant (F_3_-106) and 1 susceptible plant (F_3_-78) were detected with recombinations between the markers M9 and M10, which cover a 2.16 Mb region, respectively (Figure 2c,d). These key recombinants indicate that *ZmGHT1* is located in a physical region of 160.12–162.28 Mb in Chr8, and the recombination rate in this interval is fairly low. 

**Figure 3:**
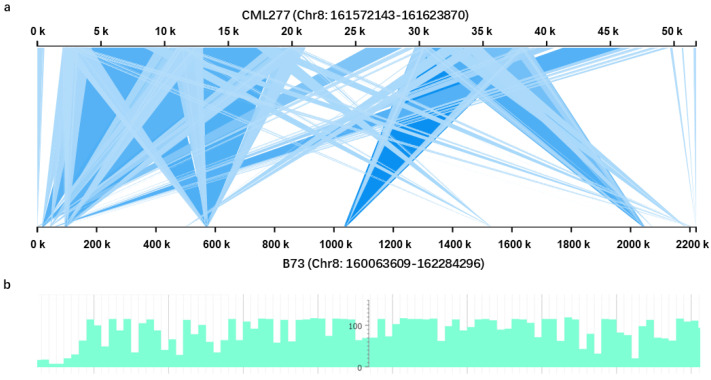
Sequence comparison between B73 and CML277 in the *ZmGHT1* locus. (**a**) Illustration of the SVs between B73 and CML277 in the *ZmGHT1* locus. (**b**) Distribution of the TEs in the *ZmGHT1* locus based on B73 genome sequence information.

To investigate the mechanism of the low recombination rate in the mapped region, four relatively long fragments (1.2–1.5 kb) were amplified from the M7 to M10 sites (Fragment 1 to Fragment 4) using L336R and L312 as templates, respectively (Table S1). Sequence comparisons were performed using the BLAST algorithm against MaizeGDB, and the results showed that the SNP and indel patterns of the PCR products of L336R are more similar to the inbred line B73, while the patterns in L312 are more similar to another sequenced inbred line, CML277 (Figures S3–S6). Further, we found that the sequence identity between L336R and B73 is 100%, while the sequence identity between L336R and CM277 is 97.1% (Table S2). In contrast, the sequence identity between L312 and CM277 is 99.6%, while the sequence identity between L312 and B73 is 97.4% (Table S2). Thus, we suspect that the sequence composition in the mapped region of L336R should be more similar to B73, while L312 is possibly similar to CML277. Interestingly, only 11.5 kb sequences exist between the M9 and M10 intervals in CML277 genome. We then retrieved the sequences between M8 and M10, where a low recombination rate was detected from B73 (2.22 Mb) and CML277 (51.73 kb), respectively. The genomic structure comparison showed that both sequence rearrangement and insertion/deletion exist between B73 and CML277 (Figure 3a). We suspect that these types of structural variations (SVs) may also exist in this region between L336R and L312, inhibiting the recombination in the *ZmGHT1* locus. We further investigated the sequence characteristics and found that relatively higher densities of transposable elements (TEs) presented in this region of the B73 genome (Figure 3b). As TE insertional polymorphisms were widely observed in the maize population [29,30], we suspect that TE insertion or deletion should be one of the reasons for the sizable SVs in the *ZmGHT1* locus.

### 2.4. Candidate Gene Prediction, Functional Annotation, and Expression Analysis

According to the annotation of the B73 reference genome in MaizeGDB, three genes, namely *Zm00001eb361900*, *Zm00001eb361920* and *Zm00001eb361930* have been predicted between M9 and M10 (Table 3). However, according to the NCBI Gnomon Gene Models [31], ten more genes were predicted (Table 3). All the thirteen genes were first functionally annotated by sequence similarity searches against GenBank. Nine were predicted as the protein coding genes and eight genes have functional annotations, among which *Zm00001eb361930* and *gene.3251669926* possibly encode class III aminotransferases, and gene.8455157 encodes a cationic amino acid transporter (Table 3).

**Figure 4:**
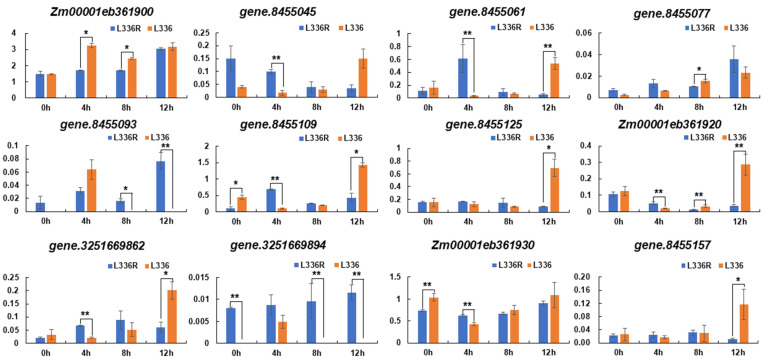
Transcript level evaluation of the candidate genes in the *ZmGHT1* locus. Gene.3251669926 was excluded due to no transcript accumulation at any time points; * indicates *p* < 0.05 and ** indicates *p* < 0.01, respectively.

We then examined the transcription patterns of the thirteen genes in response to glufosinate treatment in L336 and L336R by quantitative real time-PCR (qRT-PCR) using gene specific primers (Table S3). Except for *gene.3251669926*, all the other twelve candidate genes were detected for transcript accumulation at different time points, and *Zm00001eb361900*, *gene.8455061*, *gene.8455109*, *gene.8455125*, and *Zm00001eb361930* were relatively highly transcribed when compared to the remaining numbers (Figure 4). During the treatment with glufosinate, the temporal transcription patterns of specific genes changed and diverged significantly between L336 and L336R (Figure 4). For example, transcript levels of *Zm00001eb361900* increase after glufosinate treatment in both lines, but the transcript levels were significantly higher in L336 than those in L336R at 4 h and 8 h AGT, respectively (Figure 4). For *Zm00001eb361930*, which possibly participates in catalyzing amino groups, according to functional annotation, the transcript level was relatively higher before glufosinate treatment (0 h) in L336; however, the transcript level of L336R is statistically significantly higher than that of L336 at 4 h AGT (*p* < 0.01) (Figure 4). Although accumulation of the transcripts increased temporally, no significant differences were detected for *Zm00001eb361930* between L336 and L336R (Figure 4).

### 2.5. GS Activity Levels and Ammonia Content Determination during Glufosinate Treatment

**Figure 5:**
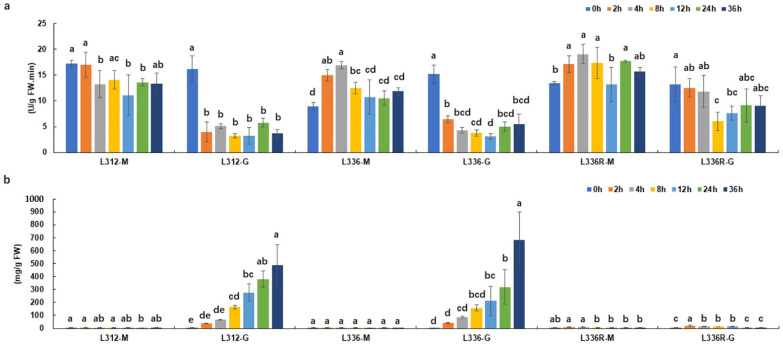
GS activity levels (**a**) and ammonia content (**b**) determination during glufosinate treatment. Different letters above each histogram bar indicate significant differences (*p* < 0.05). M: mock; G: glufosinate.

To evaluate the physiological response to glufosinate in maize, two sensitive lines L312 and L336, as well as the resistant line L336R, were treated with 1g/L glufosinate. Compared with mock treatment, GS activities of the crude extracts of L312 and L336 were rapidly and significantly decreased after glufosinate application, more than 50% reductions were observed from 2 h AGT for the both sensitive lines, and GS activity remained at roughly the same low levels during the treatment (Figure 5a). However, GS activity in L336R was less sensitive to glufosinate; the statistically significant reduction was detected until at 8 h AGT, and GS activity was slightly restored thereafter (Figure 5a).

As for ammonia, which is substrate of the reaction catalyzed by GS, no more than 10 mg ammonia per kg^−1^ fresh weight leaves were detected from untreated or water-treated plants (Figure 5b). After glufosinate treatment, the highest level of ammonia in L336R was observed at 2 h AGT, and 21.6 mg ammonia per g fresh weight (g^−1^ FW) leaves were accumulated, but the levels were then gradually restored to their original status over time (Figure 5b). For L312 and L336, rapid and significant increase in ammonia levels was observed after glufosinate treatment, up to 682.4 mg/ g^−1^ FW leaves of ammonia was detected at 36 h AGT (Figure 5b). The above data indicate that the application of glufosinate does not significantly induce in vivo ammonia accumulation in L336R.

### 2.6. Detection Efficiency Test of M9 and M10 for Identifying Functional Allele of ZmGHT1

**Figure 6:**
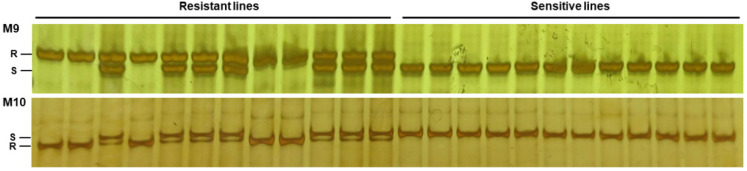
Selection efficiency validation of M9 and M10 for the ZmGHT1 functional allele. A total of 12 resistant and sensitive seedlings were randomly selected, respectively. R: resistant allele; S: sensitive allele.

A BC_2_F_2_ population including 360 seedlings, using L312 as the recurrent parent, was generated. By genotyping with M9 or M10, the same 264 seedlings were found to contain the *ZmGHT1* functional allele (Figure 6). All the 360 seedlings were then treated with 1 g/L glufosinate, and the 264 seedlings with the *ZmGHT1* functional allele survived, while the remaining 96 seedlings gradually died, indicating that M9 and M10 have high efficiencies for selecting the *ZmGHT1* functional allele. 

## 3. Discussion

Herbicides have been used as a major tool for controlling weeds in the crop fields for more than a half century [6,7]. However, the emergence of weeds resistant to widely used herbicides brings new challenges for both crop growers and scientific researchers. Considering the lower appearance rate of glufosinate resistant weeds and its potential use as an alternative herbicide to glyphosate [8,18], we screened the in-house retained maize germplasms and identified a line called L336R, which is tolerant to glufosinate (Figure 1). Further dose–response studies showed that L336R can tolerate more than two times the recommended concentration of glufosinate for weed control (Figure S2), indicating that L336R can be used as germplasm resource for breeding glufosinate tolerant maize. Genetic analysis showed that glufosinate resistance of L336R was confer by a single locus in maize Chr8, which was tentatively named *ZmGHT1* (Table 1). In the maize genome, five genes for GS1 subunits (Gln1-1 to Gln1-5) and one gene for GS2 were annotated [32,33]. We checked their chromosomal location and found that they are located on chromosomes 1, 4, 5, 9, and 10, respectively, indicating that *ZmGHT1* should not be derived from the GS gene variation, but possibly from an uncharacterized molecular target of glufosinate.

To fine map *ZmGHT1*, a biparental population was constructed. The mapped region is located in the distal region (160.12–162.28 Mb) of Chr8, which possesses a total length of 182.41 Mb sequences (Figure 2). Although a high recombination rate tends to occur in this type of subtelomeric region [34,35], extraordinarily few recombinants were detected, even using 9500 F_3_ seedlings, indicating that recombination is inhibited in the *ZmGHT1* locus. Recently, fine mapping and candidate gene identification of *Ga1* locus which, have been pursued for many years, were successfully conducted, and genomic structure analysis showed that few syntenic blocks exist between the *Ga1-S* and *ga1* genomes, which is the main reason why so few recombinant individuals were detected [36]. We therefore suspect that vast SVs between L336R and L312 in the *ZmGHT1* locus may also exist. Although the genomic sequence information is not available for L336R and L312, these two inbred lines were predicted to resemble B73 and CML277, respectively, based on sequence comparison (Figures S3–S6). Only 11.5 kb sequences were retrieved between markers M9 and M10, with a high density of TEs inserted in the non-syntenic regions of CML277 (Figure 3). This may explain the reason why so few recombinants were detected for fine mapping *ZmGHT1*. However, we cannot rule out the possible participation of other mechanisms, as studies regarding *Brassica* genomes have shown that recombination was precluded in hypermethylated regions [37].

Although phenomena of herbicide resistance have been discovered for decades, and 26 molecular target sites of herbicides have been classified by the Resistance Action Committee (https://hracglobal.com/tools/hrac-mode-of-action-classification-2022-map, accessed on 1 January 2022), new molecular targets and variations involving herbicide resistance are consistently and continuously being discovered [38,39,40,41,42,43]. In this study, eight genes located in the mapped interval were functionally annotated, among which *Zm00001eb361930* and gene.8455157, possibly encoding a class III aminotransferase and a cationic amino acid transporter, respectively, were detected for expression in the leaves during glufosinate treatment (Figure 4; Table 2). At least three types of amino acid metabolic enzymes, including EPSPSs, GSs, and the acetolactate synthases (ALSs; EC 2.2.1.6), which are crucial in the synthesis of branched-chain amino acids, have been validated as targets of different herbicides [14,17,18,44]. Specific point mutation of these enzymes endowed herbicide resistance in plants [14,18,44]. Some transferases including glutathione S-transferases (GSTs; EC 2.5.1.18) and glucosyltransferases (GTs; EC 2.4), as well as ATP-binding cassette (ABC) transporters have also been reported to be involve in detoxifying herbicides [11,41]. The above information indicates that both *Zm00001eb361930* and gene.8455157 may participate in herbicide reaction, and they are possible candidate genes for *ZmGHT1*. While assessing the expression patterns during glufosinate treatment (Figure 4), we considered *Zm00001eb361930* as the most possible candidate gene. As prominent accumulation of ammonia and a reduction in GS activity were also observed (Figure 5), indicating that the candidate gene may participate in ammonia metabolism. We found that the expression pattern of *Zm00001eb361930* is highly correlated with the fluctuation of GS activity in the resistant line L336R, that is, the reduction and restoration during glufosinate treatment (Figure 4 and Figure 5). It would thus be interesting to study the relationship between the candidate gene and ammonia catabolism and GS activity in the future.

Molecular markers M9 and M10 were used to test the selection efficiency of the *ZmGHT1* functional allele in a BC_2_F_2_ population (Figure 6). About three-fourths of the seedlings were detected to contain the *ZmGHT1* functional allele, and all of them survived after glufosinate treatment, while the remaining seedlings died. Therefore, M9 and M10 are efficient in tagging *ZmGHT1*, which should accelerate the use of this important gene in marker-assisted selection for breeding glufosinate resistant cultivars in maize.

In summary, we isolated a glufosinate resistant maize line L336R derived from natural variation, and the locus *ZmGHT1* conferring glufosinate herbicide tolerance in maize was mapped between M9 and M10 in Chr8, spanning an interval of 2.16 Mb using B73 as a reference. However, only 11.5 kb sequences were discovered in the orthologous region of CML277. Genomic structure analysis showed that SVs, including sequence rearrangement and TE insertion/deletion, are the main mechanisms for the polymorphisms. *Zm00001eb361930* located in the mapped interval is the most likely candidate gene, as determined by combining functional annotation and gene expression analysis. Our study will provide a material resource for maize herbicide-resistant breeding, with the potential to reveal a new mechanism for herbicide resistance.

## 4. Materials and Methods

### 4.1. Plant Materials and Phenotyping

In 2019, 854 maize inbred lines with 30 repetitions per line were planted in an experimental station of the University of Science and Technology Beijing in Pinggu, Beijing. At the three-leaf stage, all the 25,620 plants were screened by spraying them with 1 g/L glufosinate (Tianjin Huayu pesticide, Tianjin, China). A dose of 1 g/L was recommended by the producer for controlling normal weeds, while for perennial and resistant weeds, 2 g/L was recommended. One single plant with the glufosinate resistance potential was identified from maize inbred line L336, and it was tentatively nominated as L336R. L336R was then self-pollinated for propagation and crossed with L336 and another inbred line L312 as male parental line, respectively.

For investigating the response of L336R to glufosinate treatment and genetic inheritance, seeds of L336R, L336, and the F_1_ hybrids were germinated and grown in a greenhouse setting in a 16/8 h light/dark photoperiod with 28/22 °C temperatures. The three types of plants were treated with 1 g/L glufosinate at the three-leaf stage. To compare the fresh and dry weights of the above-ground parts, five seedlings per material were measured. Different concentrations of glufosinate ammonium (0, 1, 2, 3, 4, 5, and 8 g/L) were further used for testing the herbicide tolerance of L336R.

### 4.2. Genetic Mapping Population Construction and Fine Mapping of ZmGHT1

By crossing L336R with a glufosinate sensitive line L312 and self-pollinating the F_1_ plants, 318 F_2_ progenies were used for primary mapping. As a first step, ten F_2_ individuals of each phenotype were randomly selected for constructing a glufosinate tolerant and a sensitive bulked DNA pool, respectively. SNP polymorphism analysis was performed by using the Maize 60K SNP chip [45]. Genotyping using the Affymetrix GeneTitan platform (Thermo Fisher Scientific Affymetrix, Santa Clara, CA, USA) was performed, and the polymorphic ratios between the two bulked DNA pools were calculated in each of the 10-Mb sliding windows along the maize 10 chromosomes, and the successive region with a high polymorphic ratio was considered as the candidate region for *ZmGHT1*. 

A total of 192 F_2_ plants were randomly selected and self-pollinated to generate F_2:3_ families used for testing the segregation ratio of glufosinate tolerance; 9500 F_3_ seedlings generated from 30 F_2_ plants heterozygous for *ZmGHT1* were used for fine mapping.

To test the detection efficiency of the flanking linked markers for identifying the functional allele of *ZmGHT1*, a BC_2_F_2_ population including 360 seedlings was constructed using sensitive line L312 as the recurrent parent and L336R as the pollen donor.

### 4.3. Molecular Marker Development

The Maize 60K SNP chip assay showed that the highest polymorphic ratio occurred in the region between 154.38 Mb and 166.67 Mb in Chr8, according to the information of the B73 reference genome (AGPv5). Thus, the sequences between this interval were downloaded from MaizeGDB (https://www.maizegdb.org/, accessed on 3 August 2022), and the simple sequence repeats (SSRs) were searched using the SSR Locator program [46]; the primer pairs flanking each SSR site were designed by software Primer Premier 5.0, polymorphisms for all the molecular markers were first tested using L336R and L312 as samples, and only the co-dominant markers were retained and used for subsequent analysis.

### 4.4. Extraction of Genomic DNA and PCR Conditions

Genomic DNA was extracted from the three-leaf stage of relevant maize samples using a modified method [47]; PCR was conducted using 2×EasyTaqPCR SuperMix according to the manufacturer’s instructions (TransGen, Beijing, China). The main cycling conditions for PCR included a pre-denaturation step at 94 °C for 3 min, 35 cycles of denaturation at 94 °C for 30 s, primer annealing at 50–60 °C (adjusted depending on specific primer pairs) for 30 s, and extension at 72 °C for 45 s; a final extension was set at 72 °C for 5 min. The primer sequences for molecular markers are listed in Table 2. The primer pairs for cloning the four fragments from the M7 to M10 regions (Fragment 1 to Fragment 4), as well as the positional information based on the B73 genome, are provided in Table S1.

### 4.5. Sequence Analysis

A sequence similarity search was performed using the BLAST program [48]. Sequence alignment was performed using Clustal W [49]; sequence identity was analyzed by MatGAT2.01 [50], and further analyzed and visualized by DNAMAN version 6.0 (Lynnon Biosoft, Quebec City, QC, Canada). For visualization of the SVs of molecular markers M8 to M10 between B73 and CML277, a web-based BLAST results visualizer Kablammowas used [51].

### 4.6. RNA Extraction and Gene Expression Analysis

Seeds of L336 and L336R were sowed in plastic pots and treated with 1 g/L glufosinate at the three-leaf stage. The third leaves were harvested at 0 h, 4 h, 8 h, and 12 h time points, and total RNA was extracted using Trizol regent according to manufacturer’s protocol (Invitrogen, Waltham, MA, USA). The quality of the RNA was evaluated spectrophotometrically using NanoDrop 2000 (Thermo Scientific, Waltham, MA, USA). The reverse transcription (RT) reaction was performed using a 5× All-in-One RT MasterMix (Applied Biological Materials, Richmond, BC, Canada) in a 10 μL reaction with 500 ng of RNA as a template, following the manufacturer’s instructions. qRT–PCR analyses of the candidate genes were conducted on the QuantStudio 5 Real-Time PCR System (ABI, Carlsbad, CA, USA) using TB Green™ Premix Ex Taq™ (TaKaRa, Kyoto, Japan). *ZmActin1* was chosen and used as the internal control [52]. The data were analyzed by the 2^−ΔΔCt^ method.

### 4.7. Ammonia Accumulation Measurement

At the three-leaf stage, seedlings of L336R and L336 were sprayed with 1g/L glufosinate and distilled water (mock treatment), respectively, in a greenhouse. For ammonia extraction, the third leaves from individual plants were collected at 0, 2, 4, 8, 12, 24, and 36 h after treatment. A total of 500 mg of fresh tissues from five seedlings per sample were collected, quickly frozen in liquid nitrogen, and stored at −80 °C in an ultra-freezer until analysis. The ammonia content per g of fresh weight (mg/g^−1^ FW) leaves was determined by spectrophotometry, according to methods of Weatherburn [53], using a UV-visible spectrophotometer (Thermo Electron, Waltham, MA, USA) at a 625 nm wavelength. The assessment was conducted in triplicate.

### 4.8. GS Activity Determination

GS was extracted and measured according to the method of Ding et al. [54], using a Glutamine Synthetase (GS) Assay Kit (Solarbio, Beijing, China). In brief, 500 mg of fresh tissues from five seedlings per sample were collected, ground thoroughly in liquid nitrogen, and then diluted in 1 mL of extraction buffer. After centrifuging at 5000× *g* for 10 min, the supernatant was collected for activity measurement. The absorbance at 540 nm was recorded for the calculation of GS activity. The assessment was conducted in triplicate. The change in the absorbance value of 0.005 at 540 nm per minute g^−1^ FW leaves in the reaction system was defined as one enzyme activity unit.

### 4.9. Statistical Analysis

Data from this study were reported as mean ± standard deviation (SD). Results were subjected to analysis of variance (ANOVA) using SPSS 25.0 software (IBM SPSS Statistics for Windows, Armonk, NY, USA). Significant differences between means were assessed by Fisher’s protected least significance difference (LSD). 

## Figures and Tables

**Table 1:** The segregation ratio of F_2:3_ families and the chi-squared test.

F_2:3_ Family	Observed Frequency	Expected Frequency	χ^2^
Homozygous resistance	47	48	0.198
Heterozygous resistance	99	96	
Susceptible	46	48	

χ^2^ = 0.198 < χ^2^_0.05_ = 5.991.

**Table 2:** The molecular markers used for mapping *ZmGHT1* in this study.

Marker	Forward Primer Sequence	Reverse Primer Sequence
M1	CTGGAAAAGGAGGACTACTG	AAAGCAAGAAGTTGATATAGCC
M2	GGGACGAGTGCCAAATCAG	GGCCTTCGCAAAGAACCAA
M3	CAATCTATCGCTTGACTCATC	GCTACTACTAACTTGCCCAAAA
M4	CATACTATGTCAAAATGCATCC	GAAAACGATGATAAATGGAACT
M5	TTTCCCACAATACCAACT	GCTAGCTCTTCTGCAATC
M6	AGCCTTGTCTTTGTGGTG	GAACAGTCTGCGACTTGC
M7	CTTCCTTTAACGCCACAA	CGGCACAAGTGTTTCATCA
M8	GGAGATTAAGAGCTGCAA	ATGTTCTGGAAAGTGGTT
M9	TAGACAAGAAACCAAACAT	CAAAAGGTAAGGTGAGAC
M10	CCAACACGTGGCAGGCAG	CGCCGAGTCACCAATCCAC
M11	TATGAGTAAGTAGCGTGAAGCA	CCTATCTATTGGAAGCCTATGA

**Table 3:** Predicated genes in the *ZmGHT1* locus, their chromosomal location, and functional annotations.

Gene ID	Start	End	Strand	Functional Annotation
*Zm00001eb361900*	160124107	160128959	+	Epstein–Barr nuclear antigen
*gene.8455045*	160775402	160775752	−	No significant similarity
*gene.8455061*	160782742	160783140	−	No significant similarity
*gene.8455077*	160783139	160783441	+	No significant similarity
*gene.8455093*	161011640	161011329	+	No significant similarity
*gene.8455109*	161124578	161128321	+	Uncharacterized protein
*gene.8455125*	161128385	161129846	−	Embryogenesis transmembrane protein-like
*Zm00001eb361920*	161130057	161130464	−	Embryogenesis transmembrane protein-like
*gene.3251669862*	161493564	161495449	−	Retrovirus-related Pol polyprotein LINE-1
*gene.3251669894*	161495561	161496568	−	SH3 domain-containing protein 2
*gene.3251669926*	162029341	162030174	−	class III aminotransferase
*Zm00001eb361930*	162030458	162031088	−	class III aminotransferase
*gene.8455157*	162031992	162033874	−	Cationic amino acid transporter 1

## Data Availability

The data is contained within the article or Supplementary Materials.

## Supplementary Materials

The following supporting information can be downloaded at: https://www.mdpi.com/article/10.3390/ijms231911481/s1.
