# Supplementary material for: Genetic Analysis and Fine Mapping of ZmGHT1 Conferring Glufosinate Herbicide Tolerance in Maize (Zea mays L.)
Source: Int J Mol Sci. 2022 Sep 29;23(19):11481. doi: 10.3390/ijms231911481 (PMC9570099; doi:10.3390/ijms231911481)
Supplement: Supplementary file 1 [file ijms-23-11481-s001.zip › Figures S1 to S6.pptx]

## Slide 1
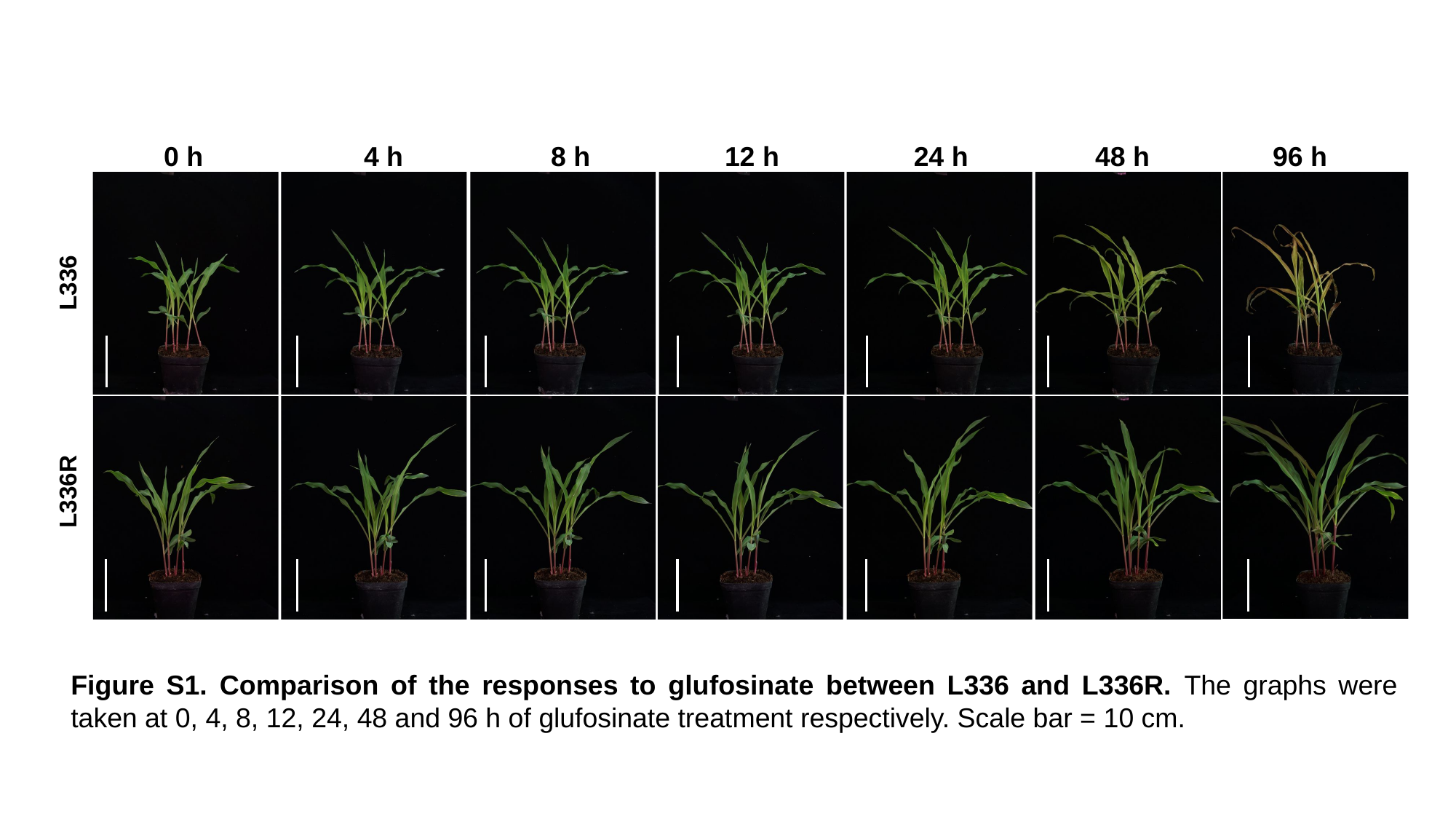

0 h
4 h
8 h
12 h
24 h
48 h
96 h
L336
L336R
Figure S1. Comparison of the responses to glufosinate between L336 and L336R. The graphs were taken at 0, 4, 8, 12, 24, 48 and 96 h of glufosinate treatment respectively. Scale bar = 10 cm.

## Slide 2
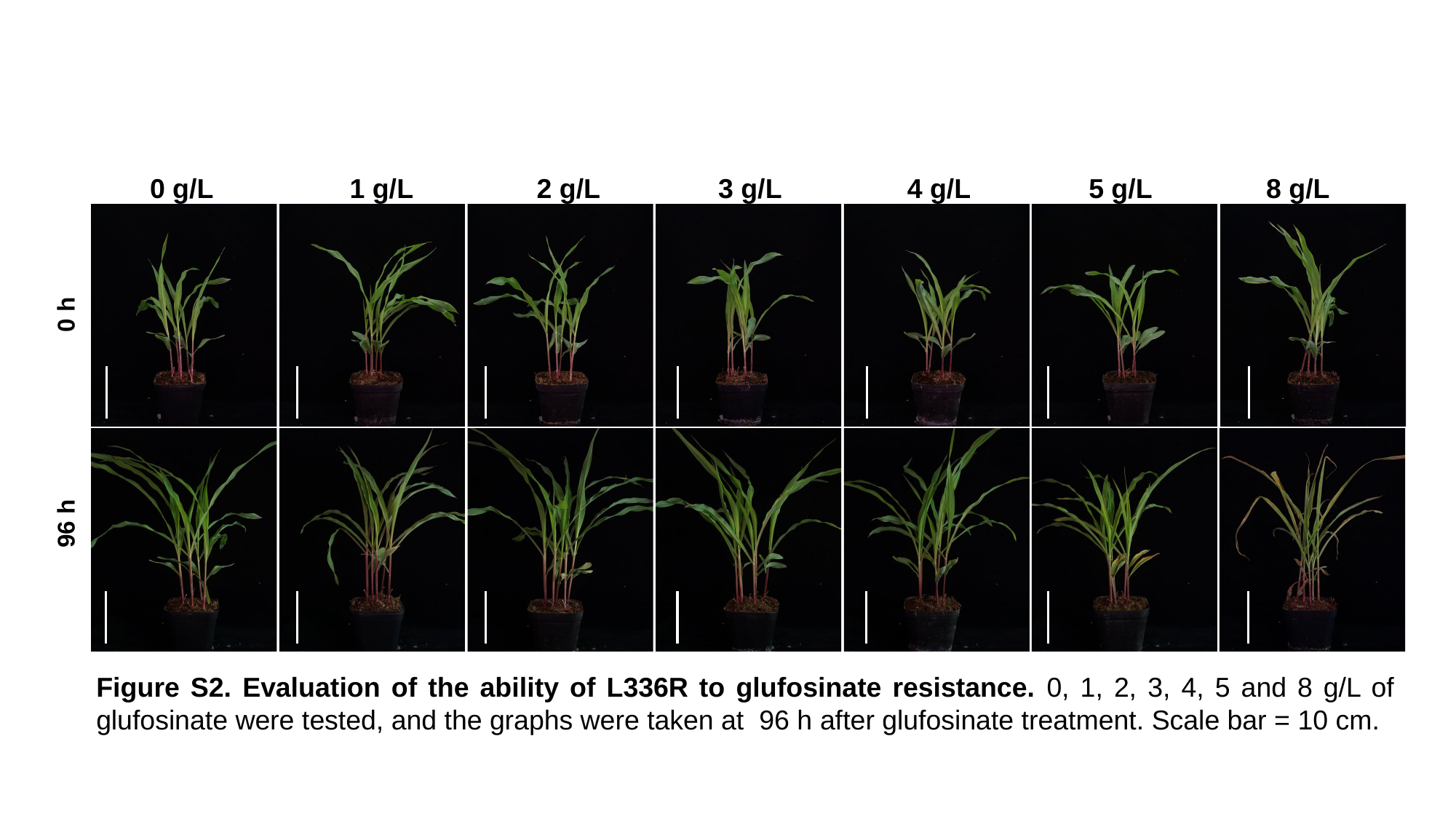

0 g/L
1 g/L
2 g/L
3 g/L
4 g/L
5 g/L
8 g/L
0 h
96 h
Figure S2. Evaluation of the ability of L336R to glufosinate resistance. 0, 1, 2, 3, 4, 5 and 8 g/L of glufosinate were tested, and the graphs were taken at 96 h after glufosinate treatment. Scale bar = 10 cm.

## Slide 3
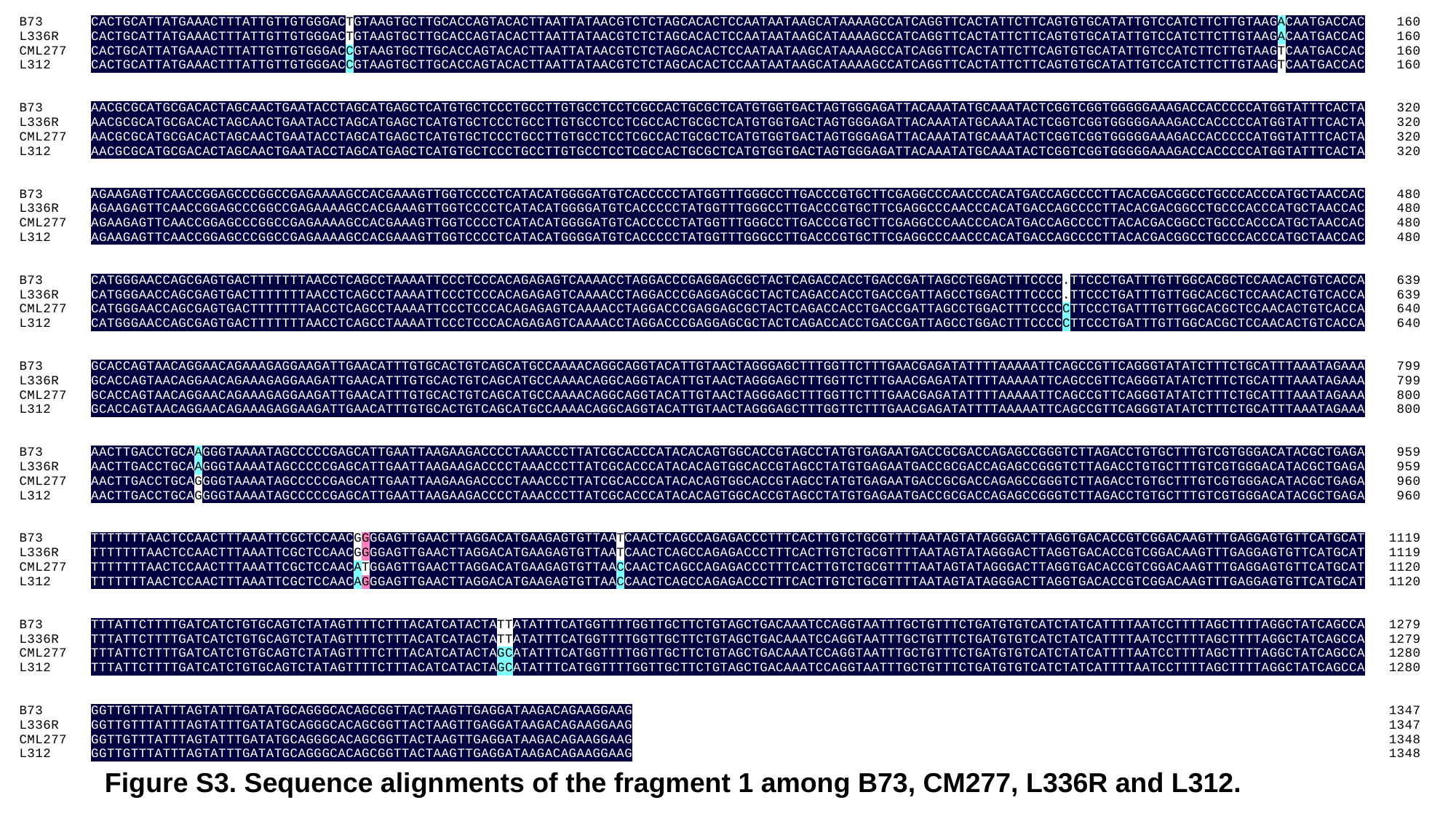

Figure S3. Sequence alignments of the fragment 1 among B73, CM277, L336R and L312.

## Slide 4
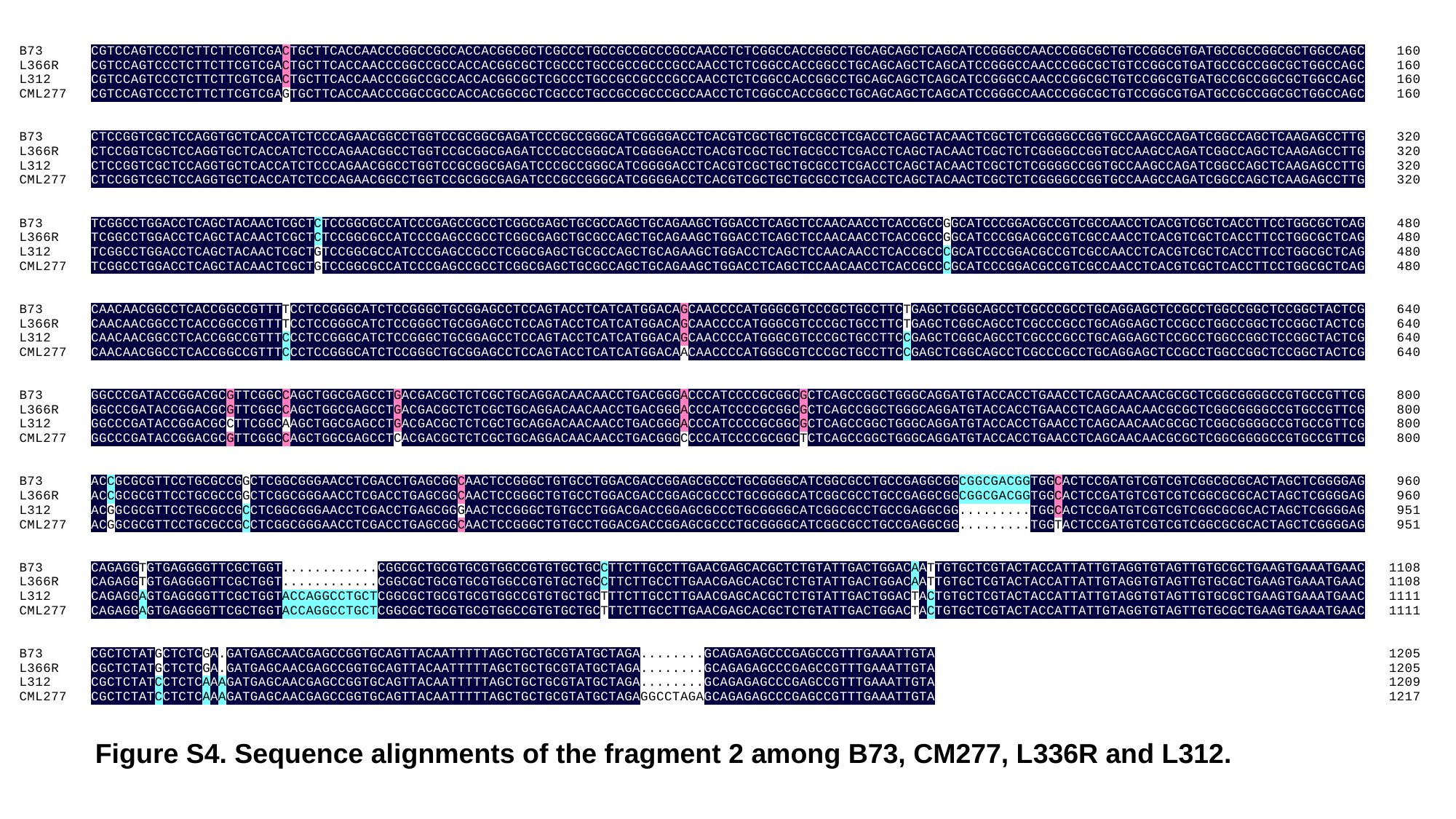

Figure S4. Sequence alignments of the fragment 2 among B73, CM277, L336R and L312.

## Slide 5
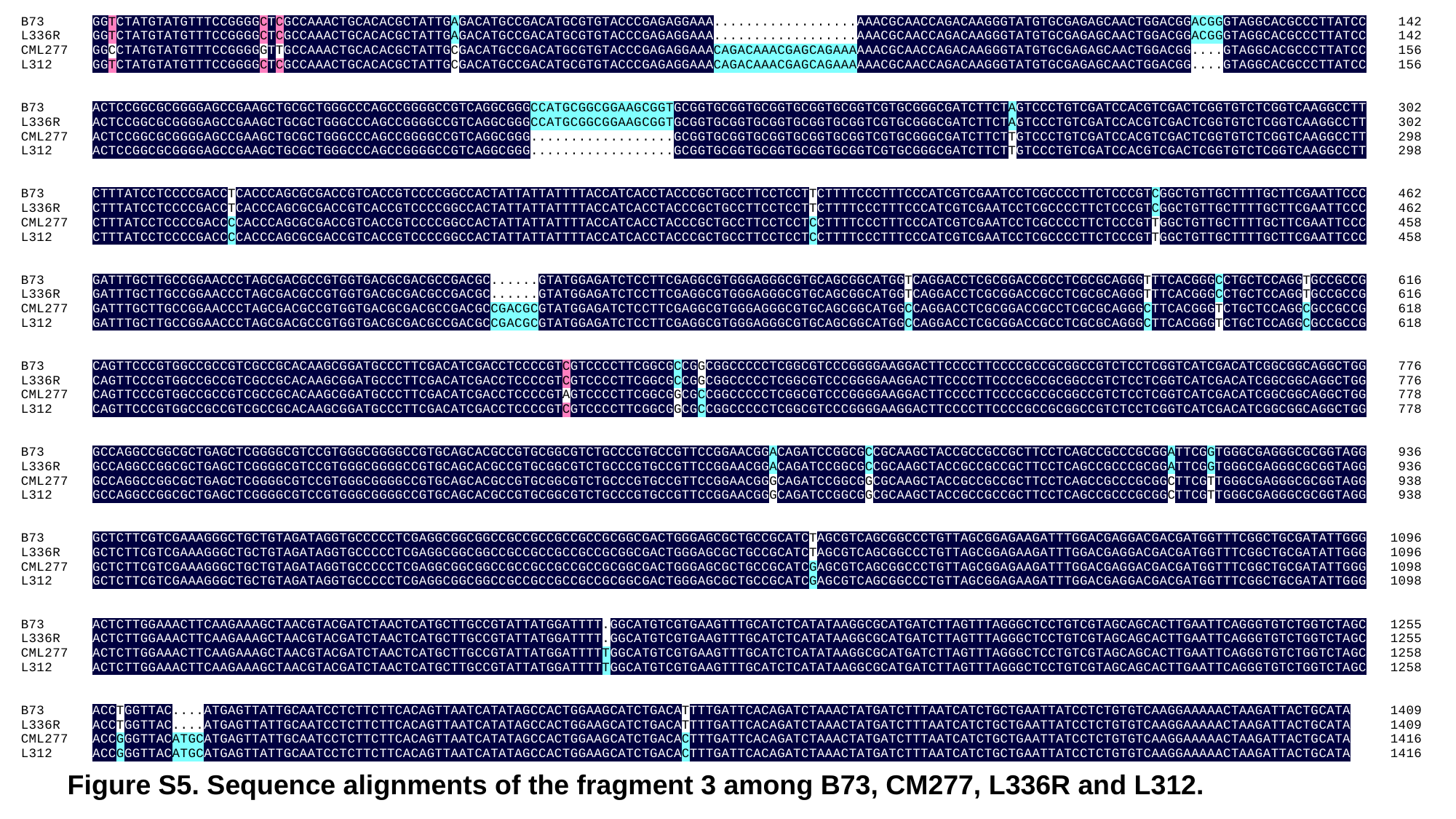

Figure S5. Sequence alignments of the fragment 3 among B73, CM277, L336R and L312.

## Slide 6
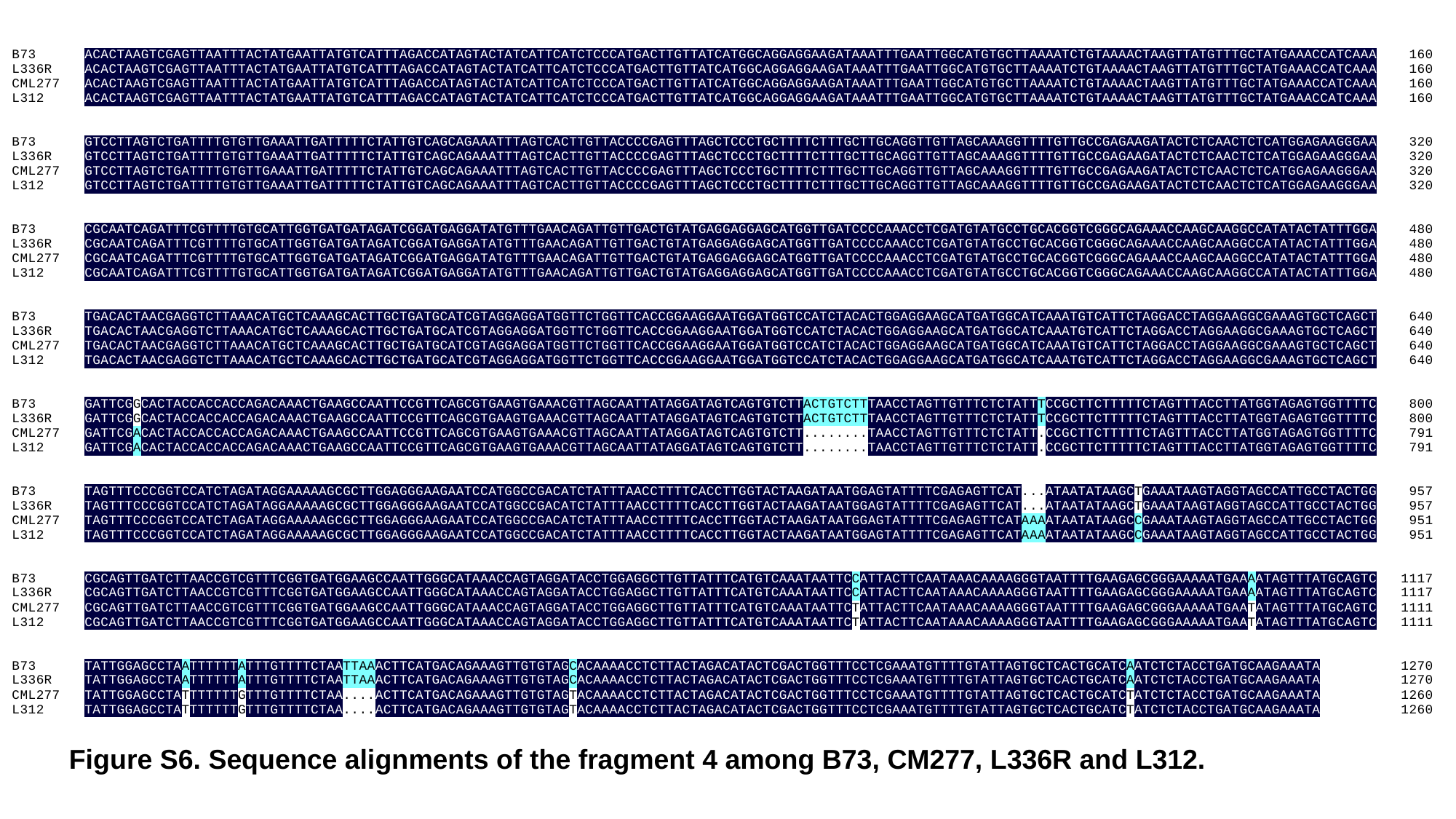

Figure S6. Sequence alignments of the fragment 4 among B73, CM277, L336R and L312.
